# Validation of an Arabic Version of the Adherence to Refills and Medications Scale (ARMS)

**DOI:** 10.3390/healthcare9111430

**Published:** 2021-10-24

**Authors:** Ghaida Alammari, Hawazin Alhazzani, Nouf AlRajhi, Ibrahim Sales, Amr Jamal, Turky H. Almigbal, Mohammed A. Batais, Yousif A. Asiri, Yazed AlRuthia

**Affiliations:** 1 Department of Clinical Pharmacy, College of Pharmacy, King Saud University, P.O. Box 2454, Riyadh 11451, Saudi Arabia; 435202385@student.ksu.edu.sa (G.A.); 435203505@student.ksu.edu.sa (H.A.); 436204108@student.ksu.edu.sa (N.A.); isales@ksu.edu.sa (I.S.); yasiri@KSU.EDU.SA (Y.A.A.); 2 Family and Community Medicine Department, College of Medicine, King Saud University, P.O. Box 3145, Riyadh 12372, Saudi Arabia; amrjamal@KSU.EDU.SA (A.J.); almogbal@yahoo.com (T.H.A.); drmohammed34@gmail.com (M.A.B.); 3 Pharmacoeconomics Research Unit, College of Pharmacy, King Saud University, P.O. Box 2454, Riyadh 11451, Saudi Arabia

**Keywords:** medication non-adherence, self-reports, validation studies, surveys and questionnaires, 12-item ARMS

## Abstract

Background: Medication non-adherence is a complex multifactorial phenomenon impacting patients with various health conditions worldwide. Therefore, its detection can improve patient outcomes and minimize the risk of adverse consequences. Even though multiple self-reported medication adherence assessment scales are available, very few of them exist in Arabic language. Therefore, the aim of this study was to validate a newly translated Arabic version of the Adherence to Refills and Medications Scale (ARMS) among patients with chronic health conditions. Methods: This is a single-center cross-sectional study that was conducted between October 10th 2018 and March 23rd 2021. ARMS was first translated to Arabic using the forward-backward translation method. The translated scale was then piloted among 21 patients with chronic health conditions (e.g., diabetes, hypertension, etc.…) to examine its reliability and comprehensibility using the test-retest method. Thereafter, the Arabic-translated ARMS was self-administered to adult patients aged ≥18 years with chronic health conditions visiting the primary care clinics of a university-affiliated tertiary care hospital in Riyadh, Saudi Arabia. Construct validity was examined using factor analysis with varimax rotation. Results: Of the 264 patients who were invited to participate, 202 (76.5%) consented and completed the questionnaire. Most of the participants were males (69.9%), married (75.2%), having a college degree or higher (50.9%), retired or unemployed (65.2%), aged ≥ 50 years (65.2%), and are diabetic (95.9%). The 12-item Arabic-translated ARMS mean score was 17.93 ± 4.90, and the scale yielded good internal consistency (Cronbach’s alpha = 0.802) and test-retest reliability (Intraclass correlation coefficient = 0.97). Two factors were extracted explaining 100% of the of the total variance (factor 1 = 52.94% and factor 2 = 47.06%). Conclusions: The 12-item Arabic version of ARMS demonstrated good validity and reliability. Therefore, it should help in the detection of medication non-adherence among Arabic-speaking patient population and minimize the risk of adverse consequences.

## 1. Introduction

Medication non-adherence is a globally recognized multifactorial phenomenon impacting patients with various health conditions [1]. The World Health Organization (WHO) defines medication adherence as “the extent to which a person’s behavior—taking medication, following a diet and/or executing lifestyle changes, corresponds with agreed recommendations from a health care provider.” [1]. Its prevalence among patients with chronic health conditions in the developed countries was estimated to be as high as 50%, and is believed to be higher in the developing countries [1,2]. In the United States, approximately half of patients with chronic illnesses are not believed to take their medications as prescribed resulting in over 100 billion United States dollars (USD) of avoidable healthcare costs [3,4]. Moreover, higher rates of hospital admissions and emergency department visits, disease progression, and poor clinical outcomes are believed to be direct consequences of poor medication adherence [3,5,6].

Even though multiple studies examined medication adherence among different patient populations worldwide, very few studies explored medication adherence levels among patients in the Middle East [7,8,9,10]. In Saudi Arabia, it was reported that up to 65% of patients with diabetes visiting primary care clinics in Al Hasa province are not adherent to their prescription medications [10]. Another questionnaire-based, cross-sectional, single-center study has estimated that 41.7% and 33% of patients with cardiovascular disease had medium and low levels of adherence to their prescription medications, respectively [9].

Multiple barriers to medication adherence have been identified in the literature [1]. Those barriers can be social (e.g., stigma associated mental illnesses) [11,12], economic factors (e.g., high copayments) [13], older age and polypharmacy [14], and poor health literacy [14]. Therefore, identifying reliable and efficient tools to assess medication adherence especially among patients with chronic health conditions became an area of research with great importance [15]. However, no gold standard medication adherence measure that can be relied upon exists so far due to the complexity of this phenomenon [16]. There are direct (e.g., face-to-face observation, and assessment of biological markers) and indirect (e.g., pill counts, and self-report questionnaires) medication adherence assessment methods with variable degrees of reliability and efficiency [16,17,18,19]. The direct medication adherence assessment tools are accurate to a great extent, however, they are somewhat invasive and impractical to use on a regular basis [19]. On the other hand, self-report questionnaires are non-invasive, less expensive, and easy to administer, albeit they are not as accurate as the direct assessment measures [20].

The studies that assessed medication adherence among Arabic-speaking patients have either used descriptive self-report questionnaires that have not been validated before or used the Arabic version of the 8-item Morisky Medication Adherence Scale (MMAS-8) which requires permission from the scale developer and administration fees [9,21]. Even though several other self-report medication adherence scales have been validated in different languages, such as Medication Adherence Questionnaire (MAQ), Brief Medication Questionnaire (BMQ), and Self-efficacy for Appropriate Medication Use Scale (SEAMS) [22,23,24,25], these scales have not been validated in Arabic.

The Adherence to Refills and Medications Scale (ARMS) is a 12-item widely used medication adherence assessment tool with proven reliability and validity among English-speaking patient population [26]. The scale was translated and validated in Turkish [27], Korean [28], Chinese [29] and Polish languages [30], among patients with chronic health conditions, such as diabetes and hypertension. However, this scale has not been validated among Arabic-speaking patients. Therefore, the aim of this study was to translate and validate ARMS into Arabic among Arabic-speaking patients with chronic health conditions.

## 2. Methods

### 2.1. Study Design

This was a single-center cross-sectional study that was conducted between October 10th 2018 and March 23rd 2021 at the primary care clinics of King Khalid University Hospital (KKUH) in Riyadh, Saudi Arabia. KKUH is a university-affiliated hospital providing primary and tertiary care to family members and relatives of King Saud University employees as well as citizens referred by the ministry of health.

### 2.2. Inclusion and Exclusion Criteria

The study included Arabic speaking adult patients aged ≥ 18 years with chronic health conditions (e.g., diabetes, hypertension, dyslipidemia, etc.) who regularly visit the primary care clinics at KKUH every six months, and have active electronic medical records (e.g., they are still eligible to receive care from the hospital). Patients without active electronic medical records, those whose native language is not Arabic, patients who do not fill their medications at KKUH pharmacy, and those with cognitive disabilities, such as dementia and Alzheimer’s diseases, were excluded.

### 2.3. Population and Data Source

Two-hundred and sixty-four patients who met the inclusion criteria were identified by reviewing their electronic medical records and ensuring that they meet the inclusion criteria. Patients’ health conditions are documented in text under the physicians’ notes. Those patients were selected systematically by selecting every 10th patient from the electronic lists of patients scheduled for regular follow-up appointments with their primary care physicians between October 2018 and March 2021 in E-Sihi database, which is the electronic health system at KKUH. The patients were recruited by three pharmacy interns who were trained to recruit patients by following a standardized protocol specifying every step on how to approach patients, explaining the purpose of the study, and inviting them to participate by asking for their consent prior to inclusion in the study.

### 2.4. Research Instrument Translation and Validation

ARMS is a 12-item self-reported medication adherence scale that was developed in English language, and consists of two subscales (adherence with filling medications and adherence with taking medications). The original scale consisted of 14 items, however, it was shortened based on the psychometric analysis conducted by the scale developers to ensure high internal consistency and reliability [26]. Adherence with filling medications subscale consists of four items, and the remaining eight items comprise the other subscale (adherence with taking medications). Each item is scored using a 4-point Likert-scale (1 = none, 2 = some, 3 = most and 4 = All). The ARMS can range from 12 to 48 with higher scores indicating poor adherence [26]. This scale has been validated among different patient populations with various chronic health conditions, such as diabetes and hypertension, and was translated to different languages [28,29,30]. Moreover, a score of ≥16 was used as a cut-off point to categorize surveyed patients into non-adherent (e.g., ≥16) and adherent (e.g., <16) [30].

In order to translate and validate the 12-item ARMS into Arabic, the permission to independently translate and validate the ARMS was obtained from the developers. Forward-backward translation method was used to translate the ARMS into Arabic. Two authors whose native language is Arabic and are proficient in English language translated ARMS into Arabic. The first draft of the translated ARMS was then reviewed for its face and content validity before being checked by a certified English language translator. Backward translation was then conducted by a native English speaker who is proficient in Arabic language, and no major issues were noticed. The pre-final version of the Arabic-ARMS was then piloted among a group of 21 patients with chronic health conditions (e.g., diabetes, hypertension, and dyslipidemia) to check its comprehensibility. The same questionnaire, which takes 10 to 15 min to complete, was re-introduced to the same group of patients two weeks later to examine its reliability using the test-retest method. No major changes were made and the final Arabic-ARMS was administered to patients who fit the inclusion criteria (Appendix A).

### 2.5. Study Variables

Sociodemographic characteristics (e.g., age, gender, monthly income, marital status, employment status, educational level, and whether the patient is enrolled in a private health insurance) were abstracted in the questionnaire. Moreover, health literacy was assessed using the single item literacy screener (SILS) developed by Morris et al. [31], and was translated and validated into Arabic by Al-Jumaili et al. [32], The SILS enquires about the need of the surveyee for someone’s help to read and understand medical or medication-related instructions with five possible responses on a Likert scale (1-never, 2- rarely, 3- sometimes, 4- often, 5- always). Those who responded with “sometimes”, “often”, and “always” are believed to have marginal or limited health literacy, and those who responded with “never” or “rarely” are believed to have adequate health literacy [31,32]. On the other hand, the medical characteristics of the participants (e.g., chronic health conditions and number of prescription medications) were also collected from the patients’ electronic health records.

### 2.6. Sample Size Estimation

Even though a sample of 100 participants is deemed sufficient to conduct most self-reported scales validation [33], the minimum sample size was estimated to be 192 subjects using GPower^®^ software version 3.1 for a medium effect size (e.g., Cohen’s d = 0.3), α = 0.05, β = 0.2, a power of 85%. Therefore, a response rate of 72.73% out of the systematically created list of 264 patients who met the inclusion criteria needs to be attained. This sample size also ensures the minimum sample needed for principal component analysis using the item to response theory [34].

### 2.7. Statistical Analysis

Descriptive statistics using frequencies and percentages, and mean ± standard deviation (SD) are shown for the participants’ characteristics and the scores of the 12-item Arabic-ARMS, respectively. In order to examine the Arabic-ARMS construct validity, principal component analysis with varimax rotation was conducted. Factors with eigenvalues greater than one were extracted [35]. Basic confirmatory factor analysis was conducted alongside the root mean square error of approximation (RMSEA) and Bentler comparative fit index (CFI) to examine the goodness of fit. Models with good fit have RMSEA ≤ 0.06 and CFI ≥ 0.9 [36]. Kaiser-Meyer-Olkin (KMO) measure was used to ensure sampling adequacy to conduct factor analysis with values above 0.5 considered satisfactory [37]. The reliability was examined using Intraclass Correlation Coefficient (ICC) for the test-retest method, and Cronbach’s alpha method. Scales with ICCs values above 0.8 [38,39] and a Cronbach’s alpha above 0.7 are generally considered reliable [40]. Convergent validity was examined using the composite reliability with a cut-off point of ≥0.6; whereas, the discriminant validity was ensured if the square root of the average variance extracted (AVE) is greater than the correlation coefficient between the different extracted factors [41,42]. Homogeneity was checked using item-total correlations, and Spearman’s correlation coefficient (rho) was calculated to examine the association between the ARMS score and different sociodemographic and medical characteristics. All statistical analyses were conducted using SAS^®^ version 9.4 (SAS institute, Cary, NC, USA).

### 2.8. Ethical Approval

The study protocol was approved by the ethics committee of King Saud University College of Medicine (E-19-3721), and all respondents verbally consented to participate in the study. No patient identifiers were collected and the data were anonymized and coded. The study adhered to the ethical principles of Helsinki declaration [43].

## 3. Results

### 3.1. Participants’ Characteristics

**Figure 1:**
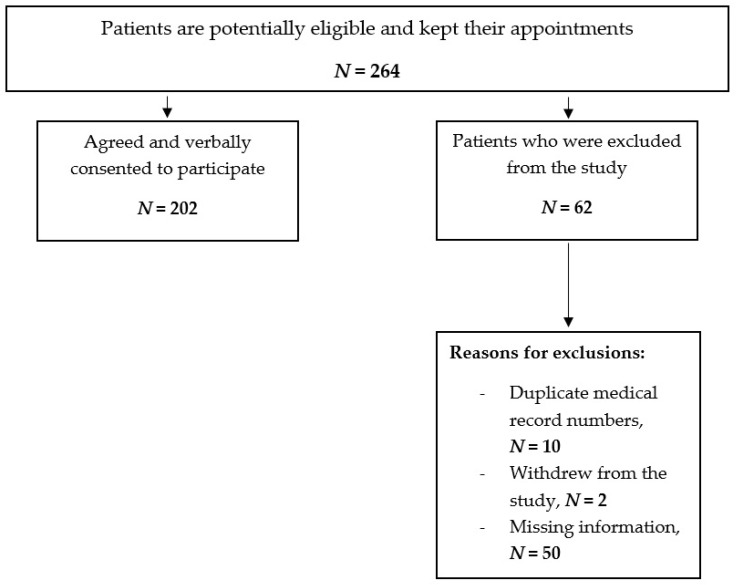
Flowchart of patient recruitment.

Out of 264 patients who fit the inclusion criteria and were invited to participate, 202 patients (76.5%) filled out the questionnaire (Figure 1). Most of the participants were males (69.9%), married (75.2%), having a college degree (e.g., a technical college diploma or bachelor’s degree) or higher (50.9%), retired or unemployed (65.2%), aged ≥ 50 years (65.2%), and 40.1% had a monthly income between 5000–10,000 SAR (USD 1333.33–USD 2666.66) as shown in Table 1. With regard to the medical characteristics of the participants, the vast majority of them (96.4%) had diabetes (e.g., 34.1% with diabetes type I and 62.3% with diabetes type II), 48% had dyslipidemia, 40% had hypertension, and 10.4% had hypothyroidism. Approximately 58% of participants reported taking six or more medications and only 12.8% had medical insurance as shown in Table 2.

### 3.2. Reliability and Internal Consistency

Using the test-retest method, the ICC for the Arabic-ARMS was 0.97 indicating good reliability. The item-total correlation coefficients for Arabic-ARMS ranged between 0.217 and 0.619, and Cronbach’s alpha values if each of the 12 items is removed ranged between 0.772 and 0.824. Overall, the Arabic-ARMS demonstrated good internal consistency with a Cronbach’s alpha of 0.802. The mean scores for the Arabic-ARMS 12 items alongside their standard deviation are shown in Table 3.

### 3.3. Factor Analysis

The KMO for sampling adequacy was 0.8 indicating sufficient sample to run factor analysis. Using principal components analysis with varimax rotation, two factors were revealed with eigenvalues greater than one as shown in Table 4. The first factor (adherence with taking medications) consisted of eight items (e.g., item-1, item-2, item-5, item-6, item-7, item-8, item-9, and item-10) explaining 52.94% of variance and having a mean score of 12.59 and a standard deviation of 3.92. On the other hand, the second factor (adherence with filling medications) consisted of four items (e.g., item-3, item-4, item-11, and item-12) explaining 47.06% of variance and having a mean score of 6.93 and a standard deviation of 2.19. The Cronbach’s alpha values for the adherence with taking medications and adherence with filling medications subscales were 0.804 and 0.665, respectively. The two extracted factors demonstrated goodness of fit using the confirmatory factor analysis with a CFI of 0.964 and RMSEA < 0.001. The composite reliabilities for the first and second factors were 0.85 and 0.64, respectively, which ensures the convergent validity. The root square of AVEs for factors one and two were 0.66 and 0.56, which are higher than the correlation coefficient between the two factors (e.g., correlation coefficient = 0.499) indicating good level of discriminant validity.

### 3.4. Adherence Scores

The mean ± SD score of the Arabic-ARMS was 17.93 ± 4.90, and its median (Q1-Q3) score was 17.00 (15.00–20.00). Using a score of ≥16 as cut-off point to categorize participants into adherent (e.g., <16) and non-adherent (e.g., ≥16), 36.14% of the participants were considered adherent and 63.86% were non-adherent. Older age (Spearman’s rank correlation coefficient (rho) = −0.157; *p*-value = 0.025), and employed patients (rho = −0.191; *p*-value = 0.006) were less likely to be non-adherent (e.g., ARMS score of ≥16). Additionally, older age (rho = −0.207; *p*-value = 0.003) but not the employment status (rho = −0.134; *p*-value = 0.056) was associated with lower total ARMS scores. On the other hand, male patients were more likely to be non-adherent in comparison to their female counterparts (rho = 0.223; *p*-value = 0.0014). Number of prescription medications (rho = 0.021; *p*-value = 0.768), number of chronic health conditions (rho = 0.035; *p*-value = 0.626), and health literacy (rho = −0.106; *p*-value = 0.132) were not associated with higher ARMS scores. Nonetheless, health literacy was negatively associated with the total ARMS score (rho = −0.211; *p*-value = 0.002), which means that those with adequate health literacy tend to have lower rates of non-adherence in comparison to their counterparts with limited health literacy levels.

## 4. Discussion

The ARMS is a widely used self-report questionnaire that has been used to assess medication adherence among diverse patient populations, particularly among older adults [26]. Additionally, it has been validated and culturally adapted to different languages, which makes it an attractive, easy to administer, free of charge, and reliable medication adherence scale [27,28,29,30]. However, the absence of a validated Arabic version of ARMS or other widely used and valid medication adherence scales that can be administered free of charge makes it hard to assess adherence to prescription medications among the Arabic-speaking patient population [15,17,21]. Therefore, translating and validating the ARMS into Arabic, which is a language spoken by more than 400 million people [44], should facilitate the assessment of medication adherence among Arabic-speaking patient population, particularly among those with chronic health conditions, such as diabetes and hypertension, which are prevalent in the Arab world [45,46,47]. This study is to the best of our knowledge the first to validate ARMS to Arabic among a cohort of patients with chronic health conditions in Saudi Arabia. The majority of the participants had diabetes and many of them had hypertension and dyslipidemia, which represents to a great extent the characteristics of patients with chronic health conditions in Saudi Arabia [46]. Additionally, the psychometric analysis showed good reliability and construct validity of the Arabic-ARMS.

The reliability of the Arabic-ARMS was assessed by two methods (test-retest and Cronbach’s alpha methods), and in the two methods the Arabic-ARMS demonstrated good reliability [38,39,40]. The Cronbach’s alpha for the 12-item Arabic-ARMS was 0.8 which is acceptable and comparable to the original scale and the other translated versions [26,27,28,29,30,48]. In addition, the two revealed factors in the factor analysis are similar to the ones identified in the original scale. The first factor consisted of eight items that assess patients’ adherence to taking their prescription medications; whereas, the second factor consists of four items and assesses patients’ adherence to filling their prescription medications. However, the variance explained by each factor and the item loadings differed from that found in the psychometric analysis of the original scale [26]. Kripalani et al. reported two factors in the original 12-item ARMS, factor 1 consisted of eight items that evaluated adherence to taking medications correctly, had an eigenvalue of 4.209 and explained 35.1% of the variance, and factor 2, consisted of four items that evaluated the participants’ ability to refill medications on schedule, had an eigenvalue of 1.199 and explained 10.0% of the variance [26]. In this study, factor analysis identified the same number of factors identified in the original scale, however the distribution of items under the factors did not match that of the original scale. Additionally, the loadings of item number 3 and item number 9 are below cutoff point of >0.4 needed for any item to be attributed to a factor [49]. This is expected since the psychometric analyses of different validated versions of ARMS revealed different percentages of variance explained by these two factors [27,28,29]. Moreover, the Korean version identified an additional factor which represented the persistence with refilling medicines [28]. Additionally, the Chinese version omitted items 4 and 11 to accommodate the nature of their patient population, which resulted in higher Cronbach’s alpha for factor 1 (*α* = 0.90) and factor 2 (*α* = 0.77) in comparison to the original scale [29]. Therefore, making appropriate adjustments to fit the patient population may generate better estimates and help in identifying unique characteristics of the targeted patient population leading to a better understanding of the reasons behind medication non-adherence.

The ARMS was originally developed among patients with low health literacy, which makes an attractive tool to assess medication non-adherence among patients with various levels of health literacy due to its high level of comprehensibility [26]. Marginal health literacy was associated with poor adherence to prescription medications in most published research studies that explored the association between medication non-adherence and different patient sociodemographic and medical characteristics [26,29,50]. Even though patients with limited health literacy were leaning toward being non-adherent (e.g., ARMS score ≥ 16), the association between health literacy and Arabic-ARMS score was not significant. This can be due to the study’s small sample or the fact that health literacy was assessed using the SILS which is not as reliable as other longer scales, such as Test of Functional Health Literacy (TOFHL) and Rapid Estimate of Adult Literacy in Medicine (REALM) [51]. However, adequate health literacy was negatively associated with the total ARMS score, which means that patients with adequate health literacy were more likely to adhere to their prescription medications. Therefore, different cut-off points for non-adherence using ARMS score should be explored.

Even though this is the first study to the best of our knowledge that translated and validated an Arabic version of the ARMS, several limitations must be acknowledged. First, patient recruitment was planned to end by March 2020, however, the COVID-19 pandemic has resulted in many appointments being postponed. Second, this is a single center study that explored the validity of a newly Arabic translated version of ARMS among patients with chronic health conditions. Therefore, the generalizability of its findings might be limited. Furthermore, since we included every 10th patient this might introduce some selection bias. Additionally, the scale was validated among a sample of patients who are largely Saudi. Thus, cultural and dialectal differences may make this Arabic translated version of ARMS not as comprehensible to non-Saudi patients as well as other Saudi patients from different geographic regions who were not represented in this study. Nonetheless, the scale was translated using Arabic terms that are understandable to anyone speaking the language. In addition, non-response bias cannot be excluded since not all patients who were invited to participate consented to participate and completed the questionnaire. Furthermore, the associations between different patient characteristics (e.g., beliefs about medications, patient medical and sociodemographic characteristics) and the ARMS score were not examined. In addition, the Arabic version of ARMS was not validated against other well-known medication adherence measures, such as pill count and medication possession ratio (MPR).

## 5. Conclusions

The findings of this study demonstrated good validity and reliability of the Arabic-ARMS, which makes it a feasible and accessible scale to many healthcare providers and researchers to assess medication adherence among patients with various health conditions. Future research should examine different cut-off points for non-adherence as well as cross-check the results with other Arabic translated scales, such as MMAS-8. Furthermore, the psychometric properties of the Arabic-ARMS should be examined among larger samples of patients with various chronic health conditions, and in different healthcare settings and cultures. Moreover, the Arabic version of ARMS should be validated against other medication adherence measures, such as pill count. Additionally, examining the relationship between different predictors of medication non-adherence (e.g., depression, beliefs about medications, polypharmacy, and older age) and the Arabic version of ARMS score should be conducted.

## Figures and Tables

**Table 1:** Study participants’ sociodemographic characteristics (*n* = 202).

**Gender**	
Male	141 (69.8)
Female	61 (30.2)
**Age group (years)**	
18–25	22 (10.8)
26–33	11 (5.4)
34–41	14 (6.9)
42–49	23 (11.3)
50–57	34 (16.8)
58–65	58 (28.7)
66–73	31 (15.3)
≥74	9 (4.4)
**Marital status**	
Single	30 (14.8)
Married	152 (75.2)
Divorced	7 (3.4)
Widowed	13 (6.4)
**Educational level**	
No official education	24 (11.8)
Completed few years of elementary school	8 (3.9)
Elementary school diploma	13 (6.4)
Middle school diploma	15 (7.4)
Secondary school diploma or equivalent (industrial or commercial diplomas)	39 (19.3)
Post-secondary school diploma or technical college	15 (7.4)
University degree	76 (37.6)
Graduate degree or equivalent (masters, doctorate, medical fellowship)	12 (5.9)
**Employment status**	
Government sector employee	51 (25.2)
Private sector employee	11 (5.4)
Freelancer	8 (3.9)
Retired	70 (34.6)
Unemployed	62 (30.6)
Monthly income *	
Less than 5000	49 (24.2)
5000–10,000	81 (40.1)
10,000–15,000	37 (18.3)
15,000–20,000	22 (10.8)
More than 20,000	13 (6.4)
**Health literacy**	
Adequate	160 (79.2)
Marginal\limited	42 (20.7)

* Presented in Saudi Arabian Riyals (3.75 SAR = 1 USD).

**Table 2:** Participants’ medical characteristics (*n* = 202).

**Number of Medications Taken**	
One	14 (6.9)
Two	18 (8.9)
Three	13 (6.4)
Four	22 (10.8)
Five	18 (8.9)
Six and more	117 (57.9)
**Chronic Diseases**	
Hypertension	99 (49.01)
Hypothyroidism	21 (10.4)
Cardiovascular disease	12 (5.9)
Dyslipidemia	97 (48)
Diabetes mellitus type I	69 (34.1)
Diabetes mellitus type II (no insulin use)	122 (60.4)
Diabetes mellitus type II (with insulin use)	4 (1.9)
Psychiatric disorders	3 (1.4)
Pulmonary diseases	15 (7.4)
Other	26 (12.9)
**Number of chronic health conditions**	
1–2	113(55.94)
3–4	80(39.60)
5–6	9(4.45)
**Medical insurance**	
Yes	26 (12.8)
No	176 (87.1)

Values are presented as *n* (%) unless otherwise specified.

**Table 3:** Item Analysis of Arabic-ARMS.

	Mean ± SD	Item–Total Correlation	Cronbach’s Alpha if Item Removed
1. How often do you forget to take your medicine?	1.50 ± 0.74	0.510	0.78
2. How often do you decide not to take your medicine?	1.29 ± 0.63	0.532	0.78
3. How often do you forget to get prescriptions filled?	1.35 ± 0.61	0.473	0.78
4. How often do you run out of medicine?	1.72 ± 0.89	0.459	0.78
5. How often do you skip a dose of your medicine before you go to the doctor?	1.25 ± 0.61	0.524	0.78
6. How often do you miss taking your medicine when you feel better?	1.29 ± 0.64	0.529	0.78
7. How often do you miss taking your medicine when you feel sick?	1.31 ± 0.74	0.528	0.78
8. How often do you miss taking your medicine when you are careless?	1.28 ± 0.65	0.612	0.77
9. How often do you change the dose of your medicines to suit your needs (like when you take more or less pills than you’re supposed to)?	1.66 ± 0.78	0.385	0.80
10. How often do you forget to take your medicine when you are supposed to take it more than once a day?	1.38 ± 0.69	0.572	0.77
11. How often do you put off refilling your medicines because they cost too much money?	1.58 ± 0.82	0.363	0.79
12. How often do you plan ahead and refill your medicines before they run out? *	2.25 ± 1.22	0.217	0.82

* This item was reverse-coded.

**Table 4:** Factor Analysis of the Arabic-ARMS (Varimax rotation method).

	Factor 1	Factor 2
% variance explained	52.94	47.06
Items		
1. How often do you forget to take your medicine?	0.808	
2. How often do you decide not to take your medicine?	0.588	
3. How often do you forget to get prescriptions filled?		0.337
4. How often do you run out of medicine?		0.639
5. How often do you skip a dose of your medicine before you go to the doctor?	0.607	
6. How often do you miss taking your medicine when you feel better?	0.648	
7. How often do you miss taking your medicine when you feel sick?	0.646	
8. How often do you miss taking your medicine when you are careless?	0.803	
9. How often do you change the dose of your medicines to suit your needs (like when you take more or less pills than you’re supposed to)?	0.338	
10. How often do you forget to take your medicine when you are supposed to take it more than once a day?	0.755	
11. How often do you put off refilling your medicines because they cost too much money?		0.402
12. How often do you plan ahead and refill your medicines before they run out?		0.446

## Data Availability

The data are available upon reasonable request from the corresponding author (Yazed AlRuthia).
